# Establishing baseline absolute risk of subsequent fracture among adults presenting to hospital with a minimal-trauma-fracture

**DOI:** 10.1186/s12891-020-3161-4

**Published:** 2020-02-28

**Authors:** Steven A. Frost, Ayano Kelly, Julia Gaudin, Lynette Mc Evoy, Carol Wilson, Lynda Marov, Carlos El Haddad, Jacqueline Center, John A. Eisman, Tuan V. Nguyen, Geraldine Hassett

**Affiliations:** 1 SPHERE MSK Clinical Academic Group, Sydney, Australia; 2 South Western Sydney Centre for Applied Nursing Research, Ingham Institute of Applied Medical Research, Sydney, NSW Australia; 3 Western Sydney University, Sydney, Australia; 4 Faculty of Medicine, UNSW Sydney, Sydney, Australia; 5 Garvan Institute of Medical Research, Darlinghurst, Australia; 6 Centre for Applied Nursing Research, Ingham Institute of Applied Medical Research, South Western Sydney Local Health District (SWSLHD), Sydney, Australia; 7 Liverpool Hospital, Sydney, Australia; 8 Bankstown-Lidcombe Hospital, Sydney, Australia; 9 Campbelltown Hospital, Sydney, Australia; 10 St Vincent’s Hospital, Sydney, Australia; 11 School of Medicine, University of Notre Dame Australia, Sydney, Australia; 12 Visiting Professor, Care and Public Health Research Institute, Maastricht University Medical Center, Maastricht, Netherlands; 13 University of Technology Sydney, Sydney, NSW Australia

## Abstract

**Background:**

One in three women and one in five men are expected to experience a minimal-trauma-fracture after the age of 50-years, which increases the risk of subsequent fracture. Importantly, timely diagnosis and optimal treatment in the form of a fracture liaison service (FLS), has been shown to reduce this risk of a subsequent fracture. However, baseline risk of subsequent fracture among this group of FLS patients has not been well described. Therefore, this study aims to estimate absolute risk of subsequent fracture, among women and men aged 50-years or more, presenting to hospital with a minimal-trauma-fracture.

**Methods:**

Women and men aged 50-years or more with a minimal-trauma-fracture, presenting to hospitals across the South Western Sydney Local Health District between January 2003 and December 2017 were followed to identify subsequent fracture presentations to hospital. Absolute risk of subsequent fracture was estimated, by taking into account the competing risk of death.

**Results:**

Between January 2003 and December 2017–15,088 patients presented to the emergency departments of the five hospitals in the SWSLHD (11,149, women [74%]), with minimal-trauma-fractures. Subsequent fractures identified during the follow-up period (median = 4.5 years [IQR, 1.6–8.2]), occurred in 2024 (13%) patients. Death during the initial hospital stay, or during a subsequent hospital visit was recorded among 1646 patients (11%). Women were observed to have 7.1% risk of subsequent fracture after 1-year, following an initial fracture; and, the risk of subsequent fracture after 1-year was 6.2% for men. After 5-years the rate among women was 13.7, and 11.3% for men, respectively. Cumulative risk of subsequent fracture when initial fractures were classified as being at proximal or distal sites are also presented.

**Conclusion:**

This study has estimated the baseline risk of subsequent fracture among women and men presenting to hospital with minimal trauma fractures. Importantly, this information can be used to communicate risk to patients deciding to attend an osteoporosis refracture prevention clinic, and highlight the need for screening, and initial of treatment when indicated, once a minimal-trauma-fracture has occurred.

## Background

The lifetime risk of osteoporotic fracture (from the age of 60) for a man and woman is 25 and 44%, respectively [1]. Experiencing a minimal-trauma-fracture (MTF) increases the risk of a subsequent fracture [2], and increases the risk of mortality [3, 4]. Importantly, timely diagnosis and optimal treatment has been shown to reduce the risk of subsequent fracture [5]. It is common that the elderly women and men who experienced MTF, do not receive appropriate assessment to establish the diagnosis of osteoporosis, or optimal treatment to prevent a subsequent fracture [6–8]. To address this challenge Fracture Liaison Services (FLS) [9] have been introduced to identify an initial fracture and ensure screening for the presence of osteoporosis, and when indicated initiate appropriate treatment to reduce the risk of another fracture.

There is increasing evidence that the introduction of a FLS is effective in reducing subsequent fracture rates [5, 10–12]. As a result, South Western Sydney Local Health District is in the process of establishing a FLS, locally described as *Osteoporosis Refracture Prevention* (ORP) Clinics across the local health district’s five acute public hospitals. In a similar manner to many other FLS, patients targeted by the service will be those presenting to hospital, aged 50-years or more, with a minimal-trauma-fracture [11–13]. The absolute risk of subsequent fracture among this group of FLS patients has not been well described, and in doing so, we propose should be part of the establishment of any new hospital based FLS. Therefore, this study was designed to estimate the absolute-risk of subsequent fracture among patients aged 50-years or more, presenting to hospital with a minimal-trauma-fracture, across our local health district, south west of Sydney, Australia.

## Methods

### Subjects and setting

South Western Sydney Local Health District delivers hospital services to a population of approximately a million people, through five acute public hospitals that have approximately 230,000 admissions each year. A FLS has been introduced in the main teaching hospital of the local health district in early 2018 with planned phased role out to each facility. The source population of this study is patients presenting to hospital emergency departments across the district, aged 50-years or more, with a minimal-trauma-fracture, between January 1st 2003 and December 31st 2017. We have included women and men, aged 50+ years in our study, due to this being the worldwide practice among fracture liaison services [9].In this study we have only included fractures related to a fall from a standing height, to ensure minimal trauma was involved in the fracture event.

### Ethical considerations

This project was considered by the South Western Sydney Local Health District Human Research Ethics Committee and was determined to meet the requirements of the National Statement on Ethical Conduct in Human Research (2007), and due to the use of routinely collected hospital separation data, the need for individual patient consent was waivered (SWSLHD HREC ref.: ETH03946).

### Identification of minimal trauma fracture

Fractures were identified using the hospital clinical coding data, based on emergency presentations with ICD-10-AM codes S22-S82. Fracture of the face and skull, hands, digits, foot and toes, were excluded. In terms of fractures of the spine, only fractures at the lumbar spine were included, as fractures of the thoracic and c-spine are often associated with trauma. Details of specific ICD-10-codes are given in a Supplementary Table. To ensure only fractures related to minimal-trauma were included, fractures codes needed concurrent coding of a fall from a standing height (ICD-10-AM W00–18), and fractures with concurrent coding of malignancy were excluded (M84.5). Date of subsequent fracture or death were also obtained from hospital separations (episodes of care) data to calculate follow-up.

### Statistical analysis

The primary outcome of this study was the time to subsequent fracture following an incident minimal-trauma-fracture, in the presence of the competing risk of death [14]. Initial fractures were classified as follows: hip, vertebral, major, and minor fractures. Major fractures included pelvis, distal femur, proximal tibia, ribs and sternum, and proximal humerus. Minor fractures included all remaining fractures, excluding those of the face, head or digits. Due to small numbers of hip fractures among patients aged < 60 years, and the relatively small numbers of lumbar-spine fractures, further analysis was undertaken by including hip and lumbar spine fractures with major fracture as *proximal fractures*, minor fractures are then referred to as *distal fractures*. Classification into distal and proximal groups was undertaken to follow some previous work, using these terms, as proximal fractures had previously been considered more serious in nature, compared to fractures of the distal skeleton [4].

Due to the presentation of rates of events, crude and adjusted relative risks of subsequent fracture based on sex, age, and site of initial fracture were estimated, and 95% confidence intervals (95%CI) using a Poisson error distribution [15]. The cumulative incidence of subsequent fracture in the presence of the competing risk of death, stratified by sex and initial fracture type, was estimated using the methods suggested by Kalbfliesch and Prentice [16]. This approach has two steps: [1] In the first step, Kaplan-Meier estimates are calculated of the overall survival from any event, in our case fracture and death, in other words both the event of interest and competing risk, respectively; and [2], in the second step the conditional probability of experiencing the event of interest, having avoided fracture and death, up until this point in time [17], in other words we have ensured that the risk of subsequent fracture has not been biased due to considering loss to follow-up among patients who have died, having a similar effect on the estimated absolute risk of subsequent fracture, as pateints who were alive at the end of the study period. Absolute risk based on sex, age, and site of initial fracture was estimated using the *survival* R package [18]. Verification of the proportional hazards assumption of the Cox models was based on a visual inspection of smoothed Schoenfeld residual plots [19].

## Results

Between January 2003 and December 2017 15,088 patients presented to the emergency departments of the five hospitals in the SWSLHD (11,149, women [74%]), with minimal-trauma-fractures (MTF). The characteristics of these patients, aged 50-years or more, are presented in Table 1. The average age of the MTF patients was 76-years (SD 12); the highest number of MTF fractures were classified as occurring at major sites (*n* = 5212 [35%]), followed by minor, 4778 (32%), hip 4738 (31%), and lumber spine, 360 (2%). Subsequent fractures identified during the follow-up period (median = 4.5 years [IQR, 1.6–8.2), occurred in 2024 (13%) patients. Death during the initial hospital stay was 1.6% (238/15,088), and 2.1% (42/2.024) during a subsequent fracture visit to hospital.

**Table 1:** Characteristics of patients presenting to hospital between January 2003 and December 2017 with minimal-trauma-fractures

	Women (*n* = 11,149)	Men (*n* = 3939)	Combined (*N* = 15, 088)	*p*-value
Age (yrs), mean (SD)	76 (12)	75 (12)	76 (12)	< 0.001
Initial fracture, N (%)				< 0.001
Hip	3375 (30)	1363 (35)	4738 (31)	
Lumbar spine	237 (3)	123 (3)	360 (2)	
Major	3661 (33)	2250 (57)	5212 (35)	
Minor	3876 (35)	903 (23)	4778 (32)	
Subsequent fracture, N (%)	1599 (14)	425 (11)	2024 (13)	< 0.001
Death, N (%)	1050 (9)	596 (15)	1646 (11)	< 0.001
Follow-up (yrs), median (IQR)	4.7 (1.7–8.4)	3.9 (1.3–7.6)	4.5 (1.6–8.2)	< 0.001

Note: Fractures were classified as follows: hip, vertebral, major, and minor fractures. Major fractures included pelvis, distal femur, proximal tibia, ribs and sternum, and proximal humerus. Minor fractures included all remaining fractures, excluding those of the face, head or digits

### Rates of subsequent fracture

Risk of subsequent fracture based on sex, age, and site of initial fracture are presented in Table 2. During the 15-year follow-up period, subsequent fracture rates were higher among women versus men (14.3% versus 10.8%, Rate Ratio (RR) = 1.33, 95% confidence interval (CI) 1.19, 1.48, *p* <  0.001); the highest rate of subsequent fracture in terms of age was that among those aged 70–79 years at the time of the initial fracture (14.9%); and, patients with an initial lumbar spine fracture, were observed to have the highest rate of subsequent fracture (15.3%), compared to hip, major and minor sites.

**Table 2:** Risk of subsequent fracture based on sex, age, and site of initial fracture

	Subsequent fracture(n)	Total (N)	Rate (%)	Rate Ratio (95% CI)		*p*-value ^a^
				Crude	Adjusted	
**Sex**						
Men	425	3939	10.79	1.0 (ref)	1.0 (ref)	
Women	1599	11,149	14.34	1.33 (1.19, 1.48)	1.31 (1.17, 1.46)	< 0.001
**Age group (yr)**						
50–59	202	1663	12.15	1.0 (ref)	1.0 (ref)	
60–69	327	2704	12.09	1.00 (0.84, 1.19)	1.01 (0.85, 1.20)	
70–79	544	3654	14.89	1.23 (1.04, 1.44)	1.28 (1.08, 1.50)	
80+	951	7067	13.46	1.11 (0.95, 1.29)	1.20 (1.00, 1.49)	0.005^b^
**Site of initial fracture**						
Hip	545	4,738,313	11.50	1.0 (ref)	1.0 (ref)	
Lumbar spine	55	360	15.28	1.33 (1.01, 1.75)	1.36 (1.03, 1.79)	0.031
Major	737	5,212,638	14.14	1.23 (1.11, 1.37)	1.20 (1.05, 1.38)	< 0.001
Minor	687	4778	14.38	1.25 (1.12, 1.40)	1.30 (1.15, 1.47)	< 0.001

Note: ^a^ adjusted *p*-value based on Wald’s test

^b^ adjusted p-value for trend, 95% CI = 95% confidence interval

Fractures were classified as follows: hip, vertebral, major, and minor fractures. Major fractures included pelvis, distal femur, proximal tibia, ribs and sternum, and proximal humerus. Minor fractures included all remaining fractures, excluding those of the face, head or digits

### Absolute risk of subsequent fracture

After taking into account the competing risk of death, the cumulative risk of subsequent fracture for 1-year, 3-years and 5-years post initial presentation to hospital, based on sex, and site of initial fracture (any site, proximal or distal) are presented in Table 3. These cumulative risks for various age groups are presented in Table 4. Women were observed to have 7.1% risk of subsequent fracture, at any site, within 1-year following an initial fracture; and, this risk of subsequent fracture after 1-year was 6.2% for men. After 5-years this rate, of fracture at any site, among women was 13.7, and 11.3% for men, respectively. Cumulative risk of subsequent fracture when initial fractures are classified a proximal, or distal are also presented. At 1-year both women (8.7% versus 6.3%) and men (9.2% versus 5.3%) were observed to have greater risk of subsequent fracture among those with a distal site of initial fracture, when compared to those with proximal site.

**Table 3:** Absolute risk of subsequent fracture during follow-up period, based on sex and site of initial fracture

Site of initial fracture	Time since initial fracture		
	1-year	3-years	5-years
**Women**			
Any	0.071 (0.066–0.076)	0.110 (0.104–0.116)	0.137 (0.130–0.144)
Proximal	0.063 (0.057–0.068)	0.109 (0.102–0.117)	0.140 (0.131–0.149)
Distal	0.087 (0.078–0.096)	0.112 (0.102–0.122)	0.132 (0.121–0.143)
**Men**			
Any	0.062 (0.055–0.070)	0.094 (0.085–0.104)	0.113 (0.102–0.124)
Proximal	0.053 (0.045–0.062)	0.086 (0.076–0.097)	0.106 (0.095–0.119)
Distal	0.092 (0.074–0.112)	0.122 (0.101–0.145)	0.134 (0.111–0.158)

Note: Fractures were classified as hip, vertebral, and major as proximal. Major fractures included pelvis, distal femur, proximal tibia, ribs and sternum, and proximal humerus. Distal fractures included all remaining fractures, excluding those of the face, head or digits

**Table 4:** Absolute risk of subsequent fracture during follow-up period, based on sex, age, and site of initial fracture

Site of initial fracture	Cumulative risk of subsequent fracture (95% confidence interval)		
	1-year	3-years	5-years
**Women**			
50–59 years			
Any	0.082 (0.068–0.097)	0.095 (0.080–0.111)	0.105 (0.089–0.123)
Proximal	0.056 (0.037–0.080)	0.079 (0.055–0.108)	0.100 (0.072–0.133)
Distal	0.094 (0.076–0.114)	0.103 (0.084–0.123)	0.108 (0.089–0.130)
60–69 years			
Any	0.079 (0.068–0.091)	0.107 (0.094–0.121)	0.127 (0.112–0.143)
Proximal	0.068 (0.053–0.085)	0.105 (0.085–0.126)	0.139 (0.115–0.164)
Distal	0.088 (0.072–0.105)	0.109 (0.091–0.128)	0.120 (0.101–0.140)
70–79 years			
Any	0.068 (0.060–0.078)	0.109 (0.098–0.121)	0.143 (0.130–0.157)
Proximal	0.058 (0.048–0.069)	0.105 (0.092–0.120)	0.144 (0.128–0.162)
Distal	0.087 (0.071–0.105)	0.116 (0.097–0.137)	0.140 (0.119–0.163)
80+ years			
Any	0.067 (0.060–0.074)	0.116(0.107–0.125)	0.145 (0.135–0.155)
Proximal	0.063 (0.056–0.071)	0.115 (0.105–0.125)	0.141 (0.130–0.153)
Distal	0.082 (0.066–0.101)	0.121 (0.100–0.143)	0.159 (0.135–0.185)
**Men**			
50–59 years			
Any	0.099 (0.076–0.126)	0.116 (0.090–0.145)	0.127 (0.099–0.158)
Proximal	0.073(0.046–0.108)	0.092 (0.061–0.131)	0.115 (0.078–0.160)
Distal	0.126 (0.090–0.168)	0.140 (0.101–0.185)	0.140 (0.101–0.185)
60–69 years			
Any	0.068 (0.052–0.086)	0.091 (0.072–0.113)	0.101 (0.081–0.125)
Proximal	0.051 (0.034–0.072)	0.078 (0.057–0.104)	0.092 (0.067–0.121)
Distal	0.099 (0.068–0.137)	0.115 (0.082–0.156)	0.120 (0.085–0.161)
70–79 years			
Any	0.053 (0.040–0.067)	0.088 (0.071–0.107)	0.108 (0.089–0.130)
Proximal	0.052 (0.038–0.068)	0.086 (0.067–0.107)	0.108 (0.087–0.132)
Distal	0.057 (0.030–0.096)	0.096(0.058–0.145)	0.111 (0.069–0.164)
80+ years			
Any	0.051 (0.041–0.062)	0.088 (0.075–0.103)	0.110 (0.095–0.127)
Proximal	0.049 (0.039–0.060)	0.084 (0.070–0.099)	0.104 (0.088–0.122)
Distal	0.067 (0.037–0.108)	0.125 (0.081–0.179)	0.156 (0.105–0.216)

Note: Fractures were classified as hip, vertebral, and major as proximal. Major fractures included pelvis, distal femur, proximal tibia, ribs and sternum, and proximal humerus. Distal fractures included all remaining fractures, excluding those of the face, head or digits

## Discussion

This study has described the absolute risk of subsequent fracture, among women and men, presenting to hospital with minimal-trauma-fracture. On average women and men, aged 50+ years or more, were observed to have a 7.1 and 6.2%, respectively - absolute risk of representing to hospital with a subsequent minimal-trauma-fracture within 1-year. These rates were approximately double after 5-years. Importantly, regardless of the site of the initial fracture, approximately 1 in every 10, women and men were at risk of a subsequent fracture in the next 3- to 5-years.

The results of this study confirm previous reports of the risk for subsequent fracture following an initial minimal-trauma-fracture [2, 4, 20, 21]. However, our estimates of the rates of absolute risk of subsequent fracture will varying from those from population based studies [2], due to our source population being limited to women and men who present to hospital. In particular, it has been highlighted that among studies based on fracture liaison services [5, 11–13], probably the most common of all osteoporotic fractures, that of the lumbar-spine, are to a significant extent, missed by this method of case finding but nevertheless an opportunity to implement osteoporosis management in this cohort. For example, in the context of clinical fractures of the lumbar spine, among women and men aged 60-years or more, the ratio of that to hip fractures is approximately 1.2–1.5, reported by various population based epidemiological studies [2, 22]. This fact alone would suggest the approach to capturing minimal-trauma-fractures, using hospital based data under-estimates the true burden, and may be missing an important population of women and men with osteoporosis, and ultimately a missed opportunity to prevent a subsequent fracture [23].

This study includes a large number of minimal-trauma-fractures, over a 15-year period, across a local health district that services a population of approximately a million people. And therefore, offers a good estimate of the burden of minimal-trauma-fractures, and subsequent fractures among women and men aged 50+ years presenting to hospital. However, a potential limitation of hospital separation fracture data, is that the fracture event must result in a presentation to hospital. And, as noted above in the context of clinical fractures of the lumbar spine, and has been identified among various reports of hospital based fracture liaison services [5, 11, 12, 23], the true burden of osteoporosis and the associated increased risk of fracture will be under-estimated.

An important clinical implication of this study is that we have been able to develop some estimates of the current baseline risk of subsequent fracture among patients that we plan to capture by implementing a fracture liaison service. These estimates will enable important information to be conveyed to patients who present to hospital with minimal-trauma-fractures and are deciding to commit to follow-up by an Osteoporosis Refracture Prevention (ORP) clinic. Importantly, as part of the implementation of ORP services across our local health district, we will be able to explore the expected and observed rates of representation to hospital with minimal-trauma-fractures. Given the current low rates of screening for osteoporosis (using DXA) among women and men who experience a minimal-trauma-fracture across our local health district, the data that we have obtained from this study will hopefully serve to improve the implementation of ORP services.

Future research in the context of implementing a fracture liaison service will need to improve the way in which women and men with minimal-trauma-fractures are identified. Innovative ways to ensure osteoporotic fractures of the spine are actively identified are currently needed. These important and common osteoporotic fractures of the spine, are obviously under-represented in our initial and subsequent fracture rates. Improvement in this area remains an important task of any fracture liaison service. And, once established, fracture liaison services will need to constantly assess their ability to keep patients on treatment, and ongoing monitoring of bone health.

## Conclusion

In conclusion, in the context of implementing a fracture liaison service, this study has estimated the baseline risk of subsequent fracture among women and men presenting to hospital with a minimal-trauma-fracture. Importantly, this information can be used to communicate risk to patients deciding to participate in *Osteoporosis Refracture Prevention* clinic, and highlight the need for screening, and initiation of treatment when indicated, once a minimal-trauma-fracture has occurred.

## Supplementary information

**Additional file 1 Supplementary Table.** Classification of fractures.

## Data Availability

Access to r-code and de-identified data can be arranged by contacting the first author.

## Abbreviations

FLS: Fracture liaison service
ICD-10-AM: International classification of disease, version 10, Australian modified
IQR: Inter-quartile range
MTF: Minimal trauma fracture
ORP: Osteoporosis refracture prevention
