# Supplementary material for: Establishing baseline absolute risk of subsequent fracture among adults presenting to hospital with a minimal-trauma-fracture
Source: BMC Musculoskelet Disord. 2020 Feb 28;21:133. doi: 10.1186/s12891-020-3161-4 (PMC7049191; doi:10.1186/s12891-020-3161-4)
Supplement: Supplementary file 1 — Additional file 1 Supplementary Table. Classification of fractures. [file 12891_2020_3161_MOESM1_ESM.docx]

**Supplementary Table.** Classification of fractures.

| **Site of initial fracture** | **ICD-10 code** |
| --- | --- |
| Hip | S72.0, S72.1, S72.2 |
| Lumbar spine | S32.0 |
| Major | S22, S32.1-S32.8, S42.0-S42.3, S42.7, S72.3-S72.5, S72.8, S82.0, S82.1 |
| Minor | S42.4, S42.8, S42.9, S52, S62, S72.7, S72.9, S82.2-S82.8 |
